# ANTICHOLINERGIC DRUGS: CUT-OFF FOR IMPAIRED COGNITION AND MOBILITY IN SENIORS

**DOI:** 10.1093/geroni/igz038.351

**Published:** 2019-11-08

**Authors:** Elpidio K ATTOH-MENSAH, Gilles Loggia, Remy Morello, Pascale Schumann-Bard, Pablo Descatoire, Christian Marcelli, Chantal Chavoix

**Affiliations:** 1 Normandie Univ, UNICAEN, INSERM, COMETE, 14000 Caen, France, Caen, France, Metropolitan; 2 CHU Caen, Department of Geriatrics, Caen, France, Caen, France, Metropolitan; 3 CHU de Caen, Department of Statistics and Clinical Research, 14000 Caen, France, Caen, France; 4 Normandie Univ, UNICAEN, INSERM, COMETE, 14000 Caen, France, Caen, France

## Abstract

Background and Objectives: Anticholinergic drugs are commonly prescribed in older adults despite growing evidence of their adverse outcomes. We aimed to improve knowledge about deleterious effects of anticholinergic drugs on both cognition and mobility, in particular whether there is a threshold value for the number of anticholinergic drugs or for the anticholinergic burden leading to mobility or cognitive impairment. Methods: 177 community-dwelling individuals aged 55 years or over, with a fall history in the previous year, took part in the study. Anticholinergic drugs were identified using the Anticholinergic Drug Scale (ADS), and global cognition and mobility were assessed using the Mini Mental State Examination (MMSE) and the Time-Up-and-Go (TUG) test, respectively. Results: ROC (Receiver Operating Characteristics) curve analysis indicated that consumption of a single anticholinergic drug per day was a risk factor for impaired MMSE (p < .05) and TUG scores (p < .05). There was also a cut-off of anticholinergic burden of one for impaired MMSE scores (p < .05). Logistic regressions showed that impaired cognition induced by anticholinergic drugs were independent of confounding factors including comorbidities, while impaired mobility would be influenced by age and cardiac comorbidities. Conclusion: Daily consumption of a single anticholinergic drug, regardless of its anticholinergic burden, impairs both cognition and mobility community-dwelling seniors. Alternative solutions to anticholinergic drug prescription should thus be considered whenever possible.

